# Pneumonia Again

**Published:** 1855-08

**Authors:** 


*Pneumonia again.*—While our City Association are engaged in the discussion of the pathology and treatment of pneumonia, we propose to go off in a little episode of our own on its causation, or rather its relations to other diseases.

Of all the books which have passed under our notice as reviewers, there is no one to which we have done such insufficient justice as "La Roche on Pneumonia and Malaria." Probably we did not at the time recognize the full scope of the work, and the importance of the principles it discusses. The great idea of the book is that pneumonia and autumnal fevers do not acknowledge the same causation. It seemed to us at the time, that this proposition did not need a great deal of proof, and that Dr. La Roche was absurdly learned in devoting so much time and labor to its support.

We have changed our mind. A more extensive acquaintance with southern and south-western medical opinions, has convinced us that Dr. La Roche was no Don Quixote warring against wind-mills. Very many southern writers are firm in the belief that the two diseases mentioned have very close relations.

The frequent concurrence of pneumonia and intermittent fever in epidemic form, is the principal argument of these theorists, supported, however, by many other analogies, some of them derived from treatment.

Dr. Upshur (Surgeon to the U. S. Marine Hospital, at Norfolk, Va.) gives, among others, in the Virginia Medical and Surgical Journal, a report of a case of pneumonia of the two lower lobes of the right lung. On the eighth day after the attack he was admitted to the hospital with well developed and highly febrile symptoms. Eight ounces of blood were taken by cups from over the right lung posteriorly, and he took a mixture containing tartar emetic, squills, and laudanum. The next day he was materially worse, with bad typhoid symptoms, and carb. ammon. and quinine were substituted for the previous treatment. This was followed by rapid convalescence, and Dr. Upshur thereupon remarks:

"I agree entirely with the opinion expressed by Dr. La Roche, in his recent learned work, that pneumonia and intermittent fevers are two separate and distinct diseases; but they so frequently occur together in the same individual, in the bilious fever regions, that without the free exhibition of the great anti-miasmatic quinine, we should lose more than half of our cases of pneumonia in this section of the country.

"The case just detailed is a very good type of nine-tenths of the cases of acute chest disease, which occur during the winter months, in the eastern parts of Virginia and North Carolina."

It is a fact worthy of notice, that in concurrence with the unusual amount of pneumonia which has been during the months of May and June prevalent in western New York, we have everywhere heard of intermittent fever. Our information upon this subject is, accidentally, quite extensive; and is based upon the statements of a very large number of practitioners both in city and country. It has been remarked in Buffalo that there were of late but two diseases, intermittent fever and pneumonia, and the same holds true of several counties to the east of us. Some of the localities referred to are malarial, none not. In some of them, however, would we be understood as stating that these diseases were epidemic, unless in the alluvial regions of

Wayne County. Their increased frequency, however, is admitted on all hands.

We do not propose to discuss the causation of either of these two diseases. *Per se*, they are probably entirely independent of each other. Dr. La Roche has shown most satisfactorily that pneumonia is common where fevers are seldom or never seen, that it is not necessarily prevalent where fevers are common, that the two diseases prevail in different seasons, that they appear under the influence of opposite winds, that pneumonia is of yearly occurrence, while fevers are not always so, that their altitudinal range is not the same, that the fever of acclimatization does not extend to pneumonia, and that the two are not convertible diseases.

Admitting all this to be proven, we must still recognize a relation between the two conditions which look back to their causation. The very fact that Dr. La Roche has taken all this pains to refute the idea, shows that local observers have seen enough to convince them of some such relation, to which they have given undue importance.

The effect of such a conviction on the therapeutics of pneumonia is well shown in the remark of Dr. Upshur, that *without the aid of the great anti-miasmatic quinine, they would lose one-half their cases of pneumonia in that region.*

The combination of pneumonia and autumnal fevers is probably, as Dr. La Roche reasons, one of those hybrid diseases which we occasionally see, and is not to be considered a special disorder. "The epidemic constitution" which predetermines intermittent fever, leaves also its impress on other diseases, pneumonia among the rest; and it is probable that when the connection is not distinctly noticed, the one exercises a considerable influence over the other. Our observation, and the remarks of very many physicians, lead us to suppose that (though the doctrine of identity is almost unknown in this region, and consequently practitioners have treated pneumonia without reference to the synchronous prevalence of intermittents) such has been the case this season, that pneumonia has been modified by the malarial epidemic constitution, and that as a result physicians have found it necessary, as a general thing, to be sparing in depletion, and have derived unusual benefit from the use of quinine. We have now in mind several such cases.

Our remark in relation to the use of quinine refers only to cases supposed to be laboring under miasmatic influences. In *pure* pneumonia we should consider it a very unsafe remedy. But if we accept the views of Dr. Upshur, sanctioned as they seem to be even by Dr. La Roche and by the general voice of southern opinion, it will be seen that whatever disposal we may make of the treatment of pure, sthenic pneumonia, we must except all of that very large number of cases originating under malarial influences f om the use of the lancet, and devote them to the anti-periodic effects of quinine.

The precise value of quinine in pneumonia is yet an open question. In curing the periodicity does it cure the inflammation?

Dr. McCaw, of Richmond, as quoted by Dr. La Roche, says: "Quinia does not apply itself to the cure of inflammation of a local character. It is not, in my opinion, an antiphlogistic at all. I know that some of the quininists do say that it is of great use as a sedative, even in this class of diseases—I do not think so myself—I have not found it so, certainly. I have given it many times during the progress of pneumonia and pleurisy; complicated with intermittent and remittent fever, I have always seen it cure the complication, but never the inflammation. In truth the short stimulating stage of the remedy would possibly add to the inflammation, but that its special influence over the accompanying fever, stopping the daily paroxysms of congestion which must be so pernicious to the favorable termination of the disease, amply repays you for this slight mischief."

So much by way of episode to the discussion now in progress, and from which our readers may hope to obtain a good idea of the prevalent doctrines in the matter of the pathology and treatment of pneumonia. The committee appointed will probably report at the next meeting, and we anticipate a valuable report.

